# Multi-scale habitat modelling and predicting change in the distribution of tiger and leopard using random forest algorithm

**DOI:** 10.1038/s41598-020-68167-z

**Published:** 2020-07-10

**Authors:** Tahir A. Rather, Sharad Kumar, Jamal A. Khan

**Affiliations:** 1 Department of Wildlife Sciences, Aligarh Muslim University, Uttar Pradesh, Aligarh, 202002 India; 2 The Corbett Foundation, 81-88, Atlanta Building, Nariman Point, Mumbai, Maharashtra 400021 India

**Keywords:** Ecology, Climate-change ecology, Ecological modelling

## Abstract

Tigers and leopards have experienced considerable declines in their population due to habitat loss and fragmentation across their historical ranges. Multi-scale habitat suitability models (HSM) can inform forest managers to aim their conservation efforts at increasing the suitable habitat for tigers by providing information regarding the scale-dependent habitat-species relationships. However the current gap of knowledge about ecological relationships driving species distribution reduces the applicability of traditional and classical statistical approaches such as generalized linear models (GLMs), or occupancy surveys to produce accurate predictive maps. This study investigates the multi-scale habitat relationships of tigers and leopards and the impacts of future climate change on their distribution using a machine-learning algorithm random forest (RF). The recent advancements in the machine-learning algorithms provide a powerful tool for building accurate predictive models of species distribution and their habitat relationships even when little ecological knowledge is available about the species. We collected species occurrence data using camera traps and indirect evidence of animal presences (scats) in the field over 2 years of rigorous sampling and used a machine-learning algorithm random forest (RF) to predict the habitat suitability maps of tiger and leopard under current and future climatic scenarios. We developed niche overlap models based on the recently developed statistical approaches to assess the patterns of niche similarity between tigers and leopards. Tiger and leopard utilized habitat resources at the broadest spatial scales (28,000 m). Our model predicted a 23% loss in the suitable habitat of tigers under the RCP 8.5 Scenario (2050). Our study of multi-scale habitat suitability modeling provides valuable information on the species habitat relationships in disturbed and human-dominated landscapes concerning two large felid species of conservation importance. These areas may act as refugee habitats for large carnivores in the future and thus should be the focus of conservation importance. This study may also provide a methodological framework for similar multi-scale and multi-species monitoring programs using robust and more accurate machine learning algorithms such as random forest.

## Introduction

Tigers and leopards are two large carnivore species of conservation importance occurring in sympatry across much of their range in India. The nationwide tiger census conducted by Govt. of India after every 4 years has shown a gradual increase in the tiger population across many protected areas. However, a significant proportion of the tiger population still occurs in fragmented landscapes outside the conventional protected areas^1,2^. Small-sized protected areas, increased habitat fragmentation, and high anthropogenic pressure on the remaining intact habitats increase the likelihood of tiger populations becoming more isolated and thereby restricting the potential dispersal opportunities^3^. Tigers and leopards are wide-ranging carnivores that require large tracts of connected habitats for persistence. Out of 12 regional tiger conservation landscapes (TCLs) in southern and north-east Asia, six tiger conservation landscapes of global conservation importance occur in the Indian subcontinent^4^. Overall, these landscapes provide habitat to over 50% of the estimated global population of wild tigers^5,6^.

In India, most of the tiger population is primarily restricted to the tiger reserves. A tiger reserve consists of an undisturbed core area wherein human settlements, livestock grazing, and resource utilization are prohibited under the Wildlife Protection Act, 1972. The core zone is further supplemented by the surrounding multi-use buffer zones, which act as population sinks. With better and strict conservation policies now employed by Govt. of India for the conservation of tigers and sympatric co-predators, there is an increased likelihood of tiger populations increase in the future. Consequent to this increase in the population of large carnivores within the limited habitats, the human-wildlife conflict in the future may be intense.

The fifth assessment report developed by the Inter-Governmental Panel for climate change^7^ projects the global surface temperature to exceed 1.5 °C by the end of the twenty-first century relative to 1850–1990 under all four Representative Concentration Pathway (RCP) scenarios. The report also projects the risk of climate-driven extinction for a large fraction of species during and beyond the twenty-first century. Climate change poses a new challenge for biodiversity conservation in the twenty-first century^8^ because the climate is one of the key environmental predictor variables of species distribution^9^. In response to climate change, species may shift to new geographically available climatic zones, adapt to new climatic conditions by means of phenotypic plasticity, or through genetic changes (evolution) or go locally extinct^10,11^. Many mammals across the world have already shifted their geographic ranges in recent decades^12,13^. Large mammals, particularly apex predators, are more sensitive to climate change and habitat fragmentation^14^.

Estimating the potential changes in the distribution of species under future climatic scenarios is thus important, particularly in the disturbed and fragmented landscapes which are more sensitive to the impacts of climate change. Species in their environment select habitat resources that determine their distribution across the range of spatial scales. Thus including the predictor variables at inappropriate spatial scale may lead to wrong conclusions^15^. It is therefore imperative to correctly identify the spatial scale at which the predictor variables best determine the distribution of species. Since the distribution of the species depends on the processes occurring at multiple spatial scales^16–18^, multi-scale distribution models can increase the predictive ability of the distribution models relative to single-scale models^17^. Multi-scale approaches are more informative and offer better insights into species habitat requirements^19–21^.

Several robust statistical approaches are available to model the species distribution in relation to the habitat variables^22,23^. Recently, however, machine learning algorithms such as Maximum Entropy Modelling (MaxEnt)^24,25^, Random forests^26^, Classification and Regressions Tress (CART)^27^ have been shown to outperform the traditional regression-based approaches. Cushman and Wasserman^28^ used multiple logistic regression and random forest algorithm in their study of multi-scale habitat selection of American martens (*Martes americana*). They found that the random forest outperformed the logistic regression approach. Similar studies of species distribution modeling report the superior ability of random forest in comparison to traditional regression-based algorithms^29–34^.

The traditional regression modelling approaches are strictly assumption based (e.g., normality, data independency, and additivity) and the predictor variables need to be pre specified. These model assumptions are seldom true in ecological context. In case of large number of explanatory variables, the traditional regression based approaches have tendency of overfitting the data unless some information criteria such as Akaiki Information Criterion (AIC) are employed to reduce the number of parameters. These limitations of conventional modelling approaches can be easily overcome by using more flexible, non-parametric algorithms such as random forest. Likewise, the likelihood based modelling approaches such as occupancy models cannot accommodate complex non-linear effects and interactions between predictor variables or covariates and are thus more suited for describing linear effects and simple interactions.

In this study, we used a random forest algorithm to investigate the scale-dependent habitat selection of tigers and leopards in the disturbed and human-dominated landscapes of central India, one of the important tiger conservation landscape areas of global conservation importance. We used scale optimized variables and predicted the habitat suitability models for tigers and leopards under the current and future climatic scenarios using the species occurrence data collected between 2017 and 2018. We used Environmental Niche Models (ENMs) to evaluate the patterns of niche similarity between tiger and leopard. The ENMs represent the predicted suitability of species or population in the landscape. These predicted suitabilities can be compared from different populations or species to test the underlying hypothesis about niche conservation or niche divergence^35–37^. The degree to which ecological niches between closely related species have been conserved over time has many implications in ecology and evolution of the species. Though ENMs can be used to compare the predicted environmental suitabilities between species, the statistical tests for assessing the similarities in the observed overlaps or testing the hypothesis of niche conservation between species or populations are lacking or conceptually ambiguous^37^. Many authors argue that such methodological ambiguity has led to the absence of general conclusions about niche conservation^38–40^. Second limitation in the studies comparing the niches of two species or populations stems from the fact that niche similarity is quantified in geographic space (G-space)^37,41^ rather than environmental space (E-space)^42,43^. The G-space is the geographic distribution of species represented by latitude and longitude that exists for any given time. The E-space as put by Broennimann et al.^42^ is defined by axes of chosen analysis (usually ordination such as PCA) and is bound by the maximum and minimum values of environmental variables found across the entire regions. Thus E-space may be viewed as the multidimensional space of environmental variables across the geographic space at any particular time, mostly characterized by first two principal components from principal component analysis^44^. Most popular methods of using E-space available are those of Broennimann et al.^42^ which comprises of two statistical tests. First statistical test called as niche equivalency test uses a monte-carlo resampling statistic to assess how similar two niches are and second test is a randomization test used to assess the power (of the equivalency test) to detect the significant differences in equivalency statistic and is called background test.

In this study, we use E-space based niche equivalency test statistic introduced by Brown and Carnaval^44^ based on methods proposed by Broennimann et al.^42^ and Qiao et al.^45^ to test for the significant differences in the environmental niches of tiger and leopard using R package ‘Humboldt’^44^. We use niche overlap test and niche divergence test to recognize the differences in environmental niches that emerge from the true niche divergence instead of other subtle causes such as difference in life history strategies, or simply due to the space limitations within the habitats^44^. Niche overlap test or niche equivalency test estimates the degree of similarity between the occupied niches and niche divergence test or niche background test estimates the portion of the accessible environment space shared by two species^44^. The niche overlap test determines how equivalent (or dissimilar) the occupied niches of two species are given the common environmental space in which they occur and in turn the niche divergent test determines the significant differences in the environmental space occupied by two species. The significant value of niche divergence test indicates that the niches of two species sharing common environmental space are not equivalent and thus the fundamental niches have resulted due to the divergent evolution.

## Results

### Univariate scaling

**Figure 1:**
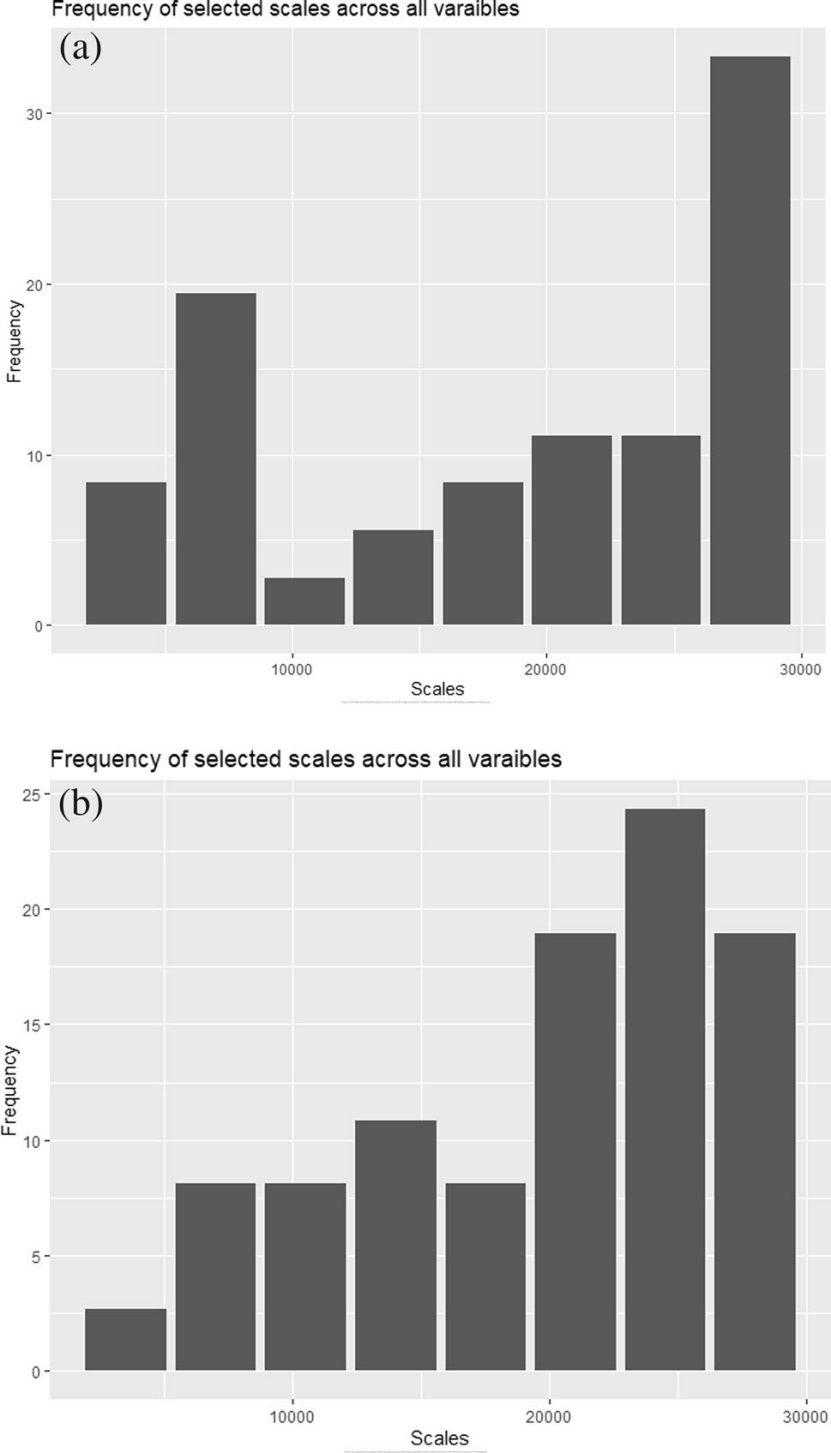
(**a**) Frequency of selected scales (in meters) across the range of predictor variables for assessing the multi-scale habitat associations of tiger. (**b**) Frequency of selected scales (in meters) across all predictor variables used to assess the multi-scale habitat associations of leopard.

A total of eight spatial scales (3,500–28,000 m) for each predictor variable except road and river density were chosen for univariate random forest modeling. Although the scales were selected across the broad range of variables, the scales at a broader spatial extent (28,000 m) had the highest frequency of selection in both the predators (Fig. 1a,b).

### Multivariate modeling

**Figure 2:**
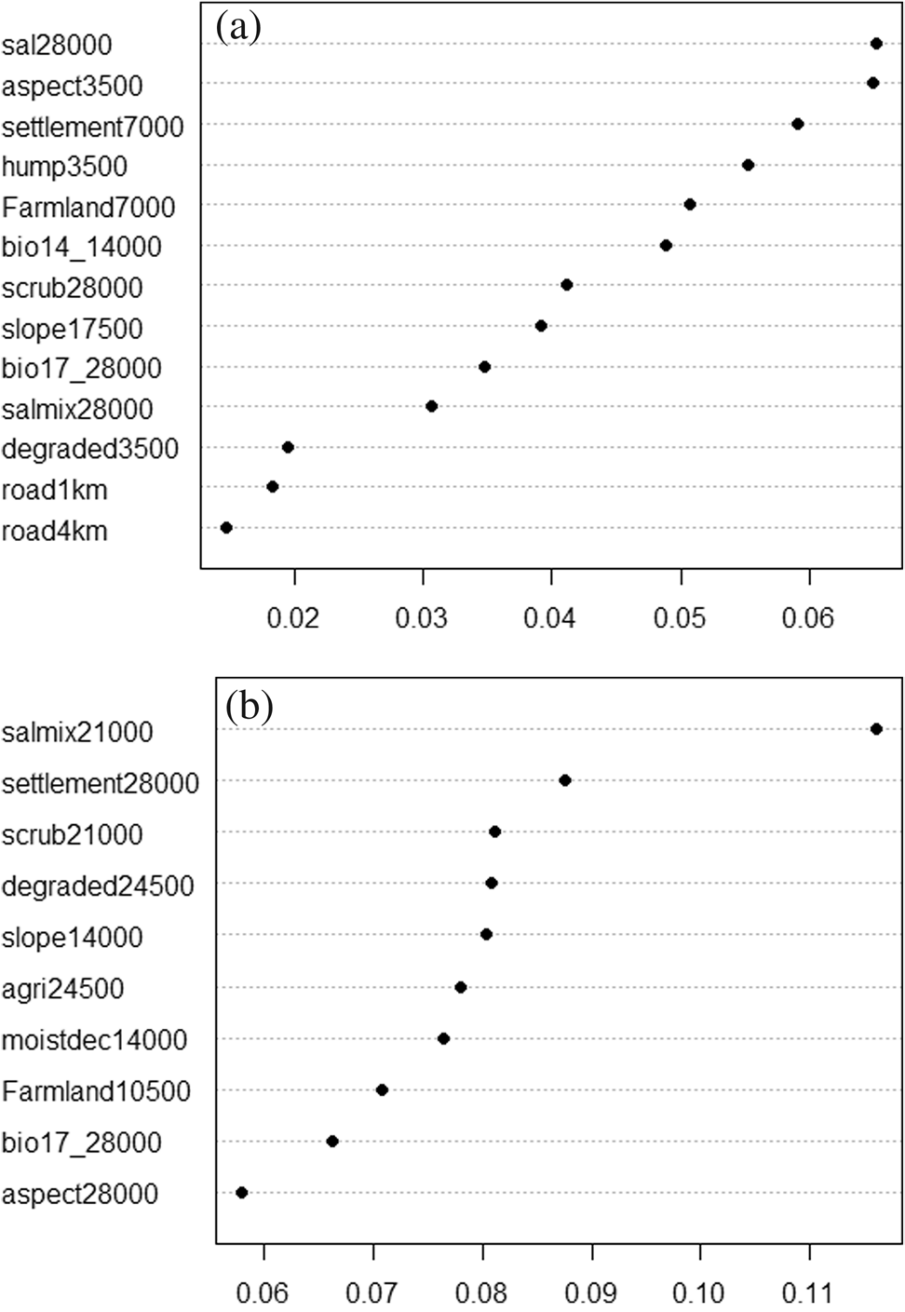
(**a**) Model improvement ratio (MIR) plot for the selected variables used in the final multiscale habitat model of tiger. Sal dominated forests at the spatial scale of 28,000 m was the most important predictor variable and road density within the focal radius of 4 km was the least important variable. The other variables are listed in order of their importance relative to sal dominated forests, with the *x*-axis indicating the relative additional model improvement when adding each successive variable. (**b**) Model improvement ratio (MIR) plot for the selected variables used in the multiscale habitat model of leopard. Sal mix forest at the spatial scale of 21,000 m was the most important predictor variable and aspect within the focal radius of 28,000 m was the least important variable. The other variables are listed in order of their importance relative to sal mix forest with the *x*-axis indicating the relative additional model improvement when adding each successive variable.

A total of 13 and 10 variables based on model improvement ratio plots (MIR) were retained in the final multivariate models of tiger and leopard (Fig. 2a,b). In the case of tigers, four variables (sal dominated forests, scrub habitats, sal mix forests and bio17) were selected at the broadest scale (28,000 m). Three variables (aspect, human population density and degraded forest patches) were selected at a small spatial scale (3,500 m), and four variables were selected at intermediate spatial scales (Human settlements, farmlands, bio14 and slope) (7,000–17,500 m). The road density was used at two spatial scales (1,000 and 4,000 m) (Fig. 2a). In the case of leopards, seven variables (sal mix forests, human settlements, scrub habitat patches, degraded forests, agriculture, bio17 and aspect) were selected at broadest scales (21,000–28,000 m) (Fig. 2b). Three of the variables (slope, moist deciduous forests and farmlands) were selected at intermediate scales (10,000–14,000 m).

### Variable importance and partial dependency plots of tiger

**Figure 3:**
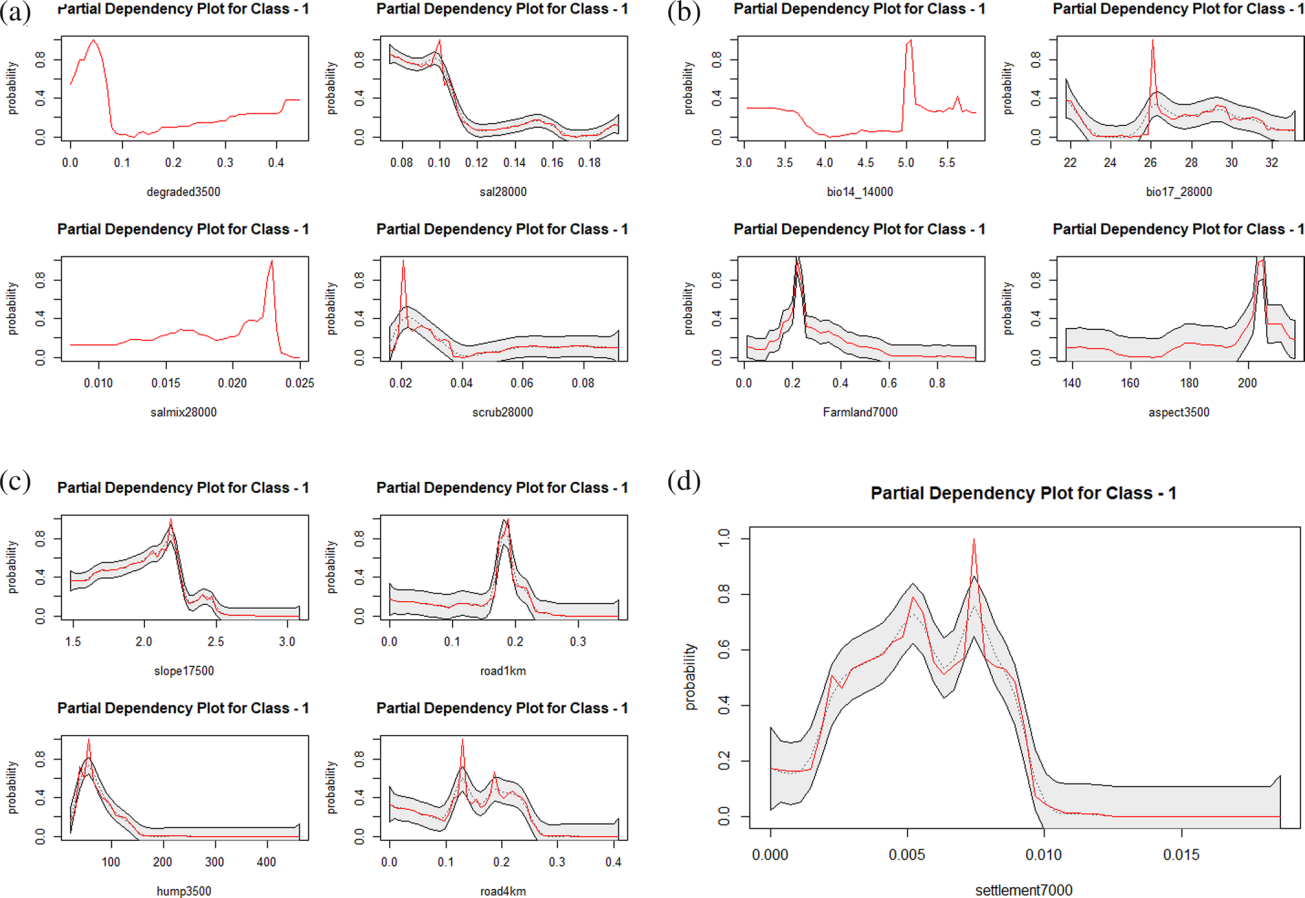
(**a**) Partial dependency plots showing the marginal effect of degraded forests, sal dominated forests, sal mix forests and scrub patches on the predicted occurrence of tiger. (**b**) Partial dependency plots showing the marginal effect of bio14, bio17, farmland and aspect on the predicted occurrence of tiger. (**c**) Partial dependency plots showing the marginal effect of slope, human population density and road density on the predicted occurrence of tiger. (**d**) Partial dependency plot showing the marginal effect of human settlement on the predicted occurrence of tiger.

The percentage of sal dominated forests at the broadest spatial scale (28,000 m) was the most important predictor variable based on MIR plots (Fig. 2a). Tiger occurrence showed a decreasing relationship with the increasing percentage of sal dominated forests at the broadest scale (Fig. 3a). Aspect at the spatial scale of 3,500 m was the second important variable (Fig. 2a). Tigers showed a strong unimodal association with the south-facing slopes (Fig. 3b). Tigers responded to the human settlements, and human population density at small spatial scales (7,000 m and 3,500 m) with the highest predicted occurrences at a lower percentage of settlements and lower human population density (Fig. 3c,d). Among bioclimatic variables, only precipitation of driest month (Bio14) and precipitation of driest quarter (Bio17) were retained as important variables in the multivariate random forest model (Fig. 2a). Tigers responded to bio14 at a small spatial scale and showed the highest predicted occurrences at higher values of the precipitation (Fig. 3b).

In contrast, bio17 was most influencing at the broadest scale. The highest occurrences of tigers were predicted at the intermediate levels of precipitation in the driest quarter (Fig. 3b). The mosaic of croplands and natural vegetation was most influencing at a small spatial scale of 7,000 m. Tigers showed the tendency to avoid the habitats with a higher percentage of farmlands (Fig. 3b). Tigers responded to the open scrub habitats at the lower percentage and broadest scale (28,000 m). Tigers chose very gentle slopes at the intermediate scale (17,500 m) with a strong unimodal relationship around 2 degrees of steepness (Fig. 3c). Tigers showed a strong unimodal preference for sal mixed forest at the broadest spatial scale (Fig. 3a). The degraded forest patches were perceived at small spatial scales and tigers showed an overall avoidance of degraded forests (Fig. 3a). There was a strong unimodal relationship between tigers and road density within 1 km, and the highest occurrences of tigers were predicted at 0.2 densities of roads within 1 km focal radii (Fig. 3c). In contrast, we observed a moderate relationship relative to the road density within 4 km focal radii (Fig. 3c).

### Variable importance and partial dependency plots of leopard

**Figure 4:**
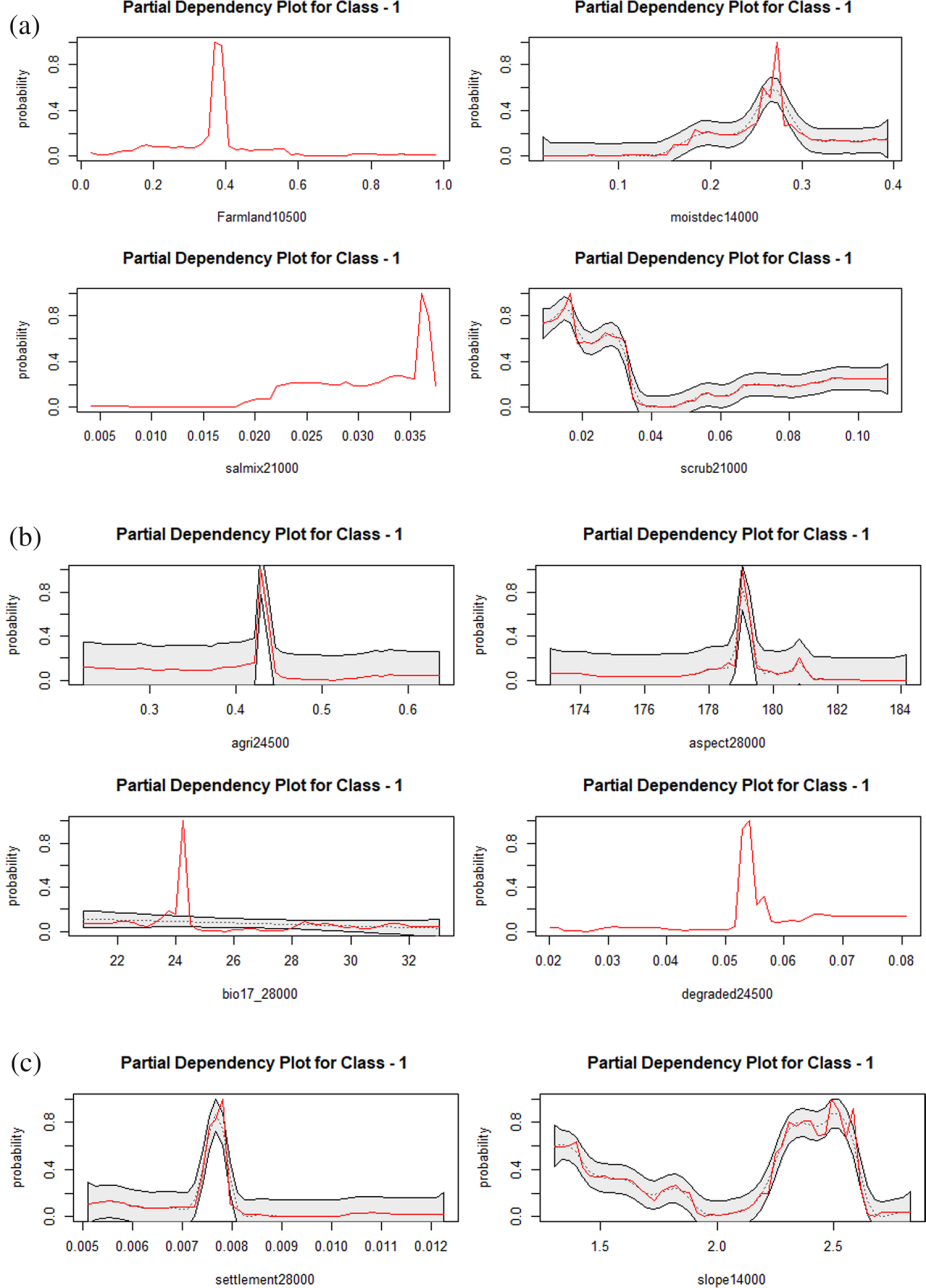
(**a**) Partial dependency plot showing the marginal effects of farmland, moist deciduous forests, sal mix forests and scrub habitats on the predicted occurrence of leopard. (**b**) Partial dependency plot showing the marginal effect of agriculture, aspect, bio17, and degraded forests on the predicted occurrence of leopard. (**c**) Partial dependency plots showing the marginal effect of human settlements and slope on the predicted occurrences of leopard.

Leopards showed a preference for the high percentage of sal mix forests at a broader spatial scale (Fig. 4a). Leopards showed a unimodal preference for moist deciduous forests at medium concentrations and intermediate spatial scale (Fig. 4a). Leopards preferred the mosaic of cropland and natural vegetation at an intermediate spatial scale (10,500 m) with the highest occurrence predicted at a lower percentage of farmland (Fig. 4a). High preference of leopard for the intermediate percentage of degraded habitats occurred at a broader spatial scale (Fig. 4b). A strong unimodal relationship existed between leopards and the percentage of agricultural patches at a broader spatial scale (Fig. 4b). The precipitation of driest quarter (bio17) had a unimodal effect on the habitat selection of leopards best explained at the broadest spatial scale (Fig. 4b). The aspect was the tenth important predictor variables influencing the habitat selection of leopards at the broadest spatial scale (28,000 m) with a high occurrence of leopards along southern slopes (Fig. 4b). A unimodal relationship was predicted at a lower percentage of human settlements at a broader spatial scale (Fig. 4c). Leopards showed a general preference for gentle slopes with the highest predicted occurrence at 2.5° (Fig. 4c).

### Distribution of tigers and leopards under current and future climatic scenarios

**Figure 5:**
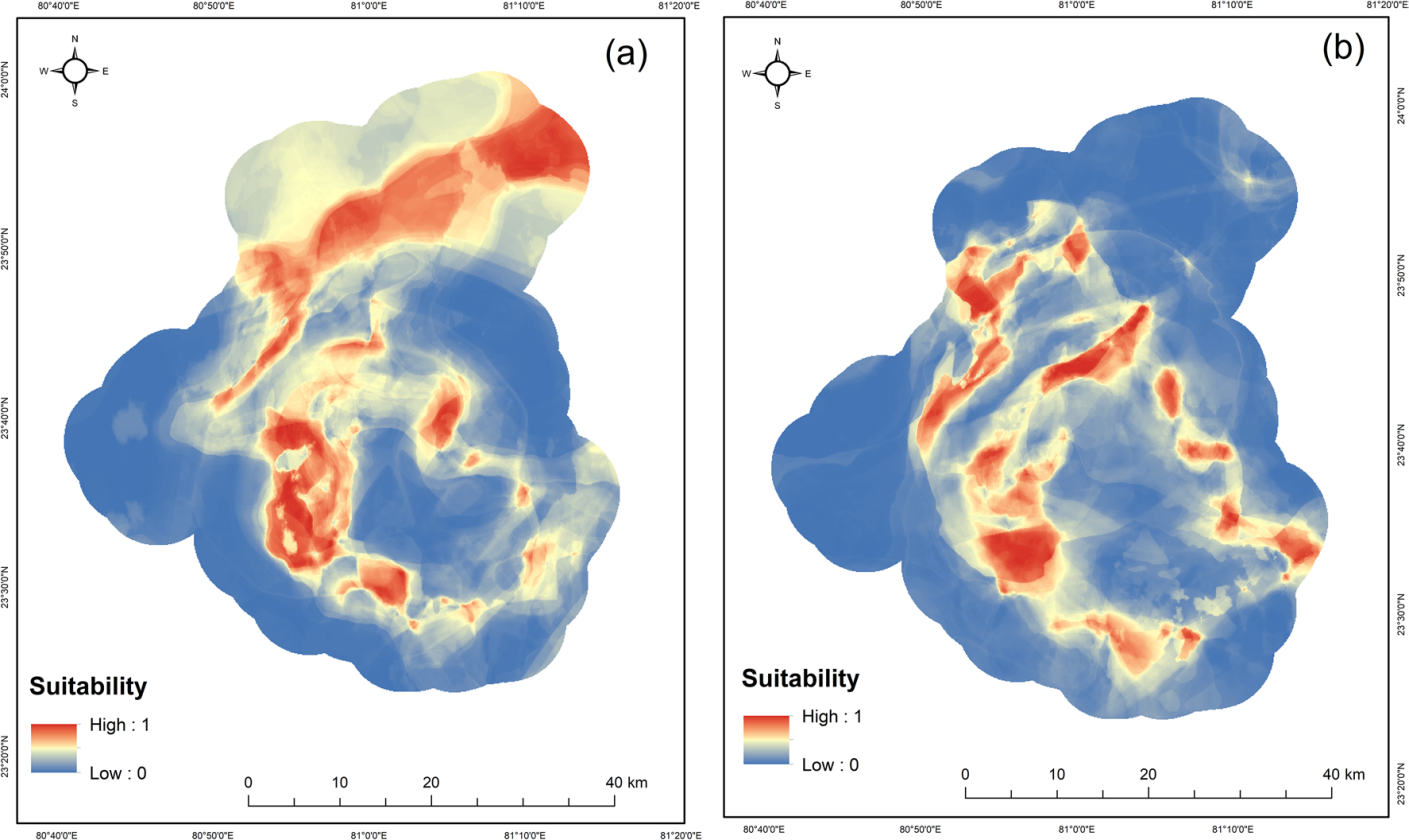
Predicted habitat suitability under current climatic scenario based on the scale optimized predictor variables using Random forest algorithm. Panel (**a**) represents the habitat suitability of tiger and panel (**b**) represents the suitability of leopard. Red color denotes high suitability and blue color indicates low suitability.

**Figure 6:**
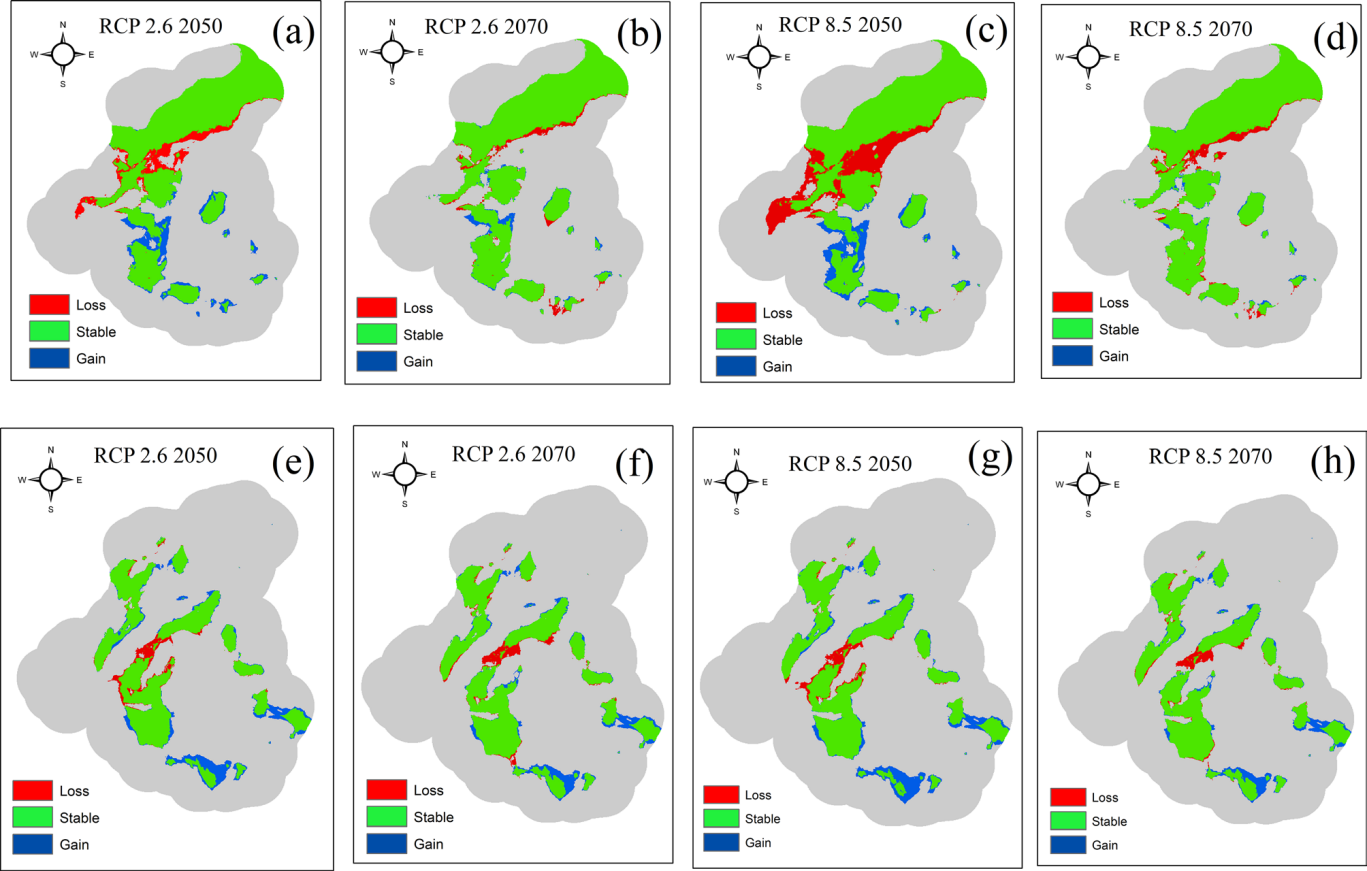
Predicted change in the distribution of tigers and leopards under future climatic scenarios. Panels (**a**) and (**b**) represent the change in distribution of tiger under RCP 2.6 scenario for the years 2050s and 2070s. Panels (**c**) and (**d**) represent the change in distribution of tiger under RCP 8.5 for the years 2050s and 2070s. Panels (**e**) and (**f**) represent the change in the distribution of leopard under RCP 2.6 Scenario for the years 2050s and 2070s and panels (**g**) and (**h**) represent the predicted change in the distribution of leopard under RCP 8.5 for the years 2050s and 2070s. The map was created using ArcGIS (v 10.3) software developed by ESRI. https://www.esri.com.

The spatial distribution of tigers and leopards was predicted using the scale optimized predictor variables selected in the process of variable importance in multiscale random forest models. The accuracy for the distribution maps of leopards was discriminately high (AUC = 0.90, TSS = 0.80) in comparison to tigers (AUC = 0.83, TSS = 0.66). A total of 65,499.17 (42.64%) and 39,770.20 (25.89%) hectares of suitable habitat exists for tigers and leopards under the current climatic scenario. The suitable area for tigers included the northern Panpatha wildlife sanctuary, which forms the core zone of the reserve and the dense sal dominated forest in the southeastern parts of the reserve (Fig. 5). For leopards, the most suitable areas included the forested areas at the edges of the core-buffer boundary of the reserve (Fig. 5). We predicted the change in the distribution of tigers and leopards under the most conservative emission pathway scenario (RCP 2.6) and worst emission scenario (RCP 8.5). Our model showed the overall loss in the suitable habitat of tigers under both the scenarios, while our model predicted overall gain in habitats for leopards (Fig. 6). The highest loss (23%) in the suitable habitat of tigers was predicted under RCP 8.5 for the years the 2050s and 11% under RCP 2.6 for the years 2050s (Table 1).

**Table 1:** Predicted change in the distribution of tigers and leopards in and around Bandhavgarh Tiger Reserve, Madhya Pradesh, India under low (RCP 2.6) and high (RCP 8.5) Representative Concentration Pathway scenarios for the timeline 2050s and 2070s using the model developed by Model for Inter-disciplinary Research on Climate change (MIROC5).

Species	Scenario	Total suitable habitat	Stable habitat (ha)	Stable habitat (%)	Gain (ha)	Gain (%)	Loss (ha)	Loss (%)	Net gain/net loss (ha)	Net gain/net loss (%)
Tiger	Current	65,499.17	65,499.17	100.00						
	RCP 2.6 (2050)	64,987.03	58,125.97	88.74	6,861.06	10.48	7,373.20	11.26	− 512.14	0.0013
	RCP 8.5 (2050)	57,764.33	50,289.49	76.78	7,474.84	11.41	15,209.68	23.22	− 7,734.84	− 0.0012
	RCP 2.6 (2070)	64,377.20	61,732.99	94.25	2,644.20	4.04	3,766.18	5.75	− 1,121.97	− 0.0001
	RCP 8.5 (2070)	61,701.48	60,226.52	91.95	1,474.95	2.25	5,272.65	8.05	− 3,797.69	− 0.0004
Leopard	Current	39,770.19	39,770.19	100						
	RCP 2.6 (2050)	43,564.73	36,745.43	92.39	6,819.30	17.14	3,024.76	7.60	3,794.54	0.0009
	RCP 8.5 (2050)	44,120.20	36,914.83	92.82	7,205.37	18.11	2,855.36	7.17	4,350.01	0.00012
	RCP 2.6 (2070)	42,152.81	36,730.46	92.35	5,422.34	13.63	3,039.73	7.64	2,382.62	− 0.0004
	RCP 8.5 (2070)	42,585.37	37,201.631	93.54	5,383.74	13.53	2,568.56	6.45	2,815.18	0.0001

The highest gain (11.41%) in the suitable habitat was also predicted under RCP 8.5 for the 2050s. The gain in suitable habitat was more pronounced relative to the loss in the habitat for leopards under both the emission scenarios (Table 1). The highest gain in habitat was predicted under RCP 8.5 (18.12%), followed by RCP 2.6 (17.15%). The amount of the habitat loss was predicted to be almost the same for leopards under all pathway scenarios.

### Niche similarity between tiger and leopard

**Figure 7:**
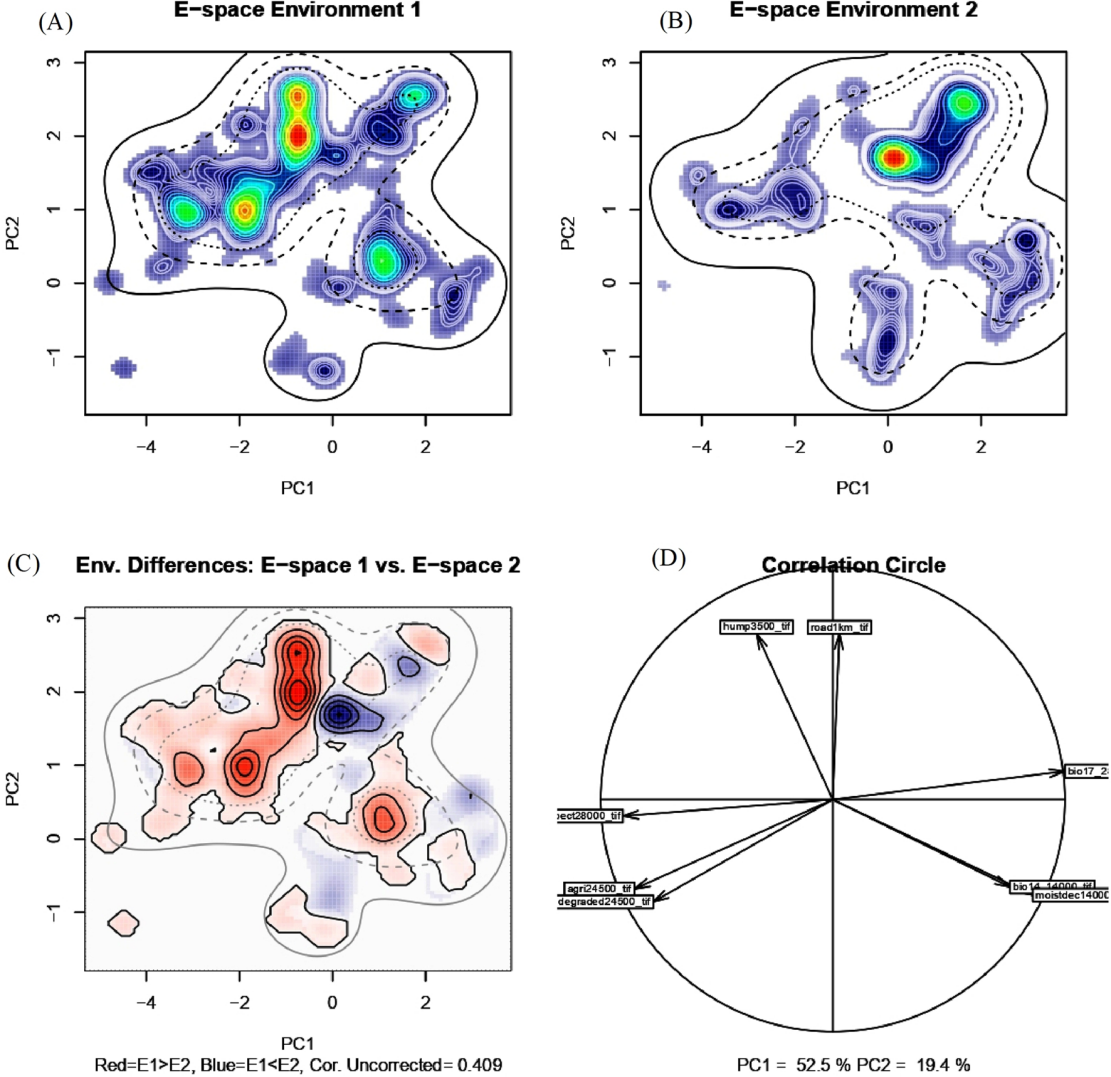
Niches of tiger and leopard in two dimensional E-space. Panels (**A**) and (**B**) represent the niches of species along first two axes of the PCA. The species occurrences are represented by kernel density isopleths, red color indicates high density and cooler color (blue) indicates low density. Solid and dotted contour lines illustrate 100% and 50% of the available background (environmental space). Panel (**C**) represents the difference in the E-space of two species and Niche E-space Correlation Index (NECI). NCEI determines if one should correct the occurrence densities of each species by the prevalence of their environments in their range for equivalency and background tests. For high NCEI (> 0.5) species occupied niches are recommended to be corrected by the frequency of E-space in accessible environments to reduce the chances of committing type 1 errors, and panel (**D**) represents the correlation circle and important principal components of the raw input data.

**Figure 8:**
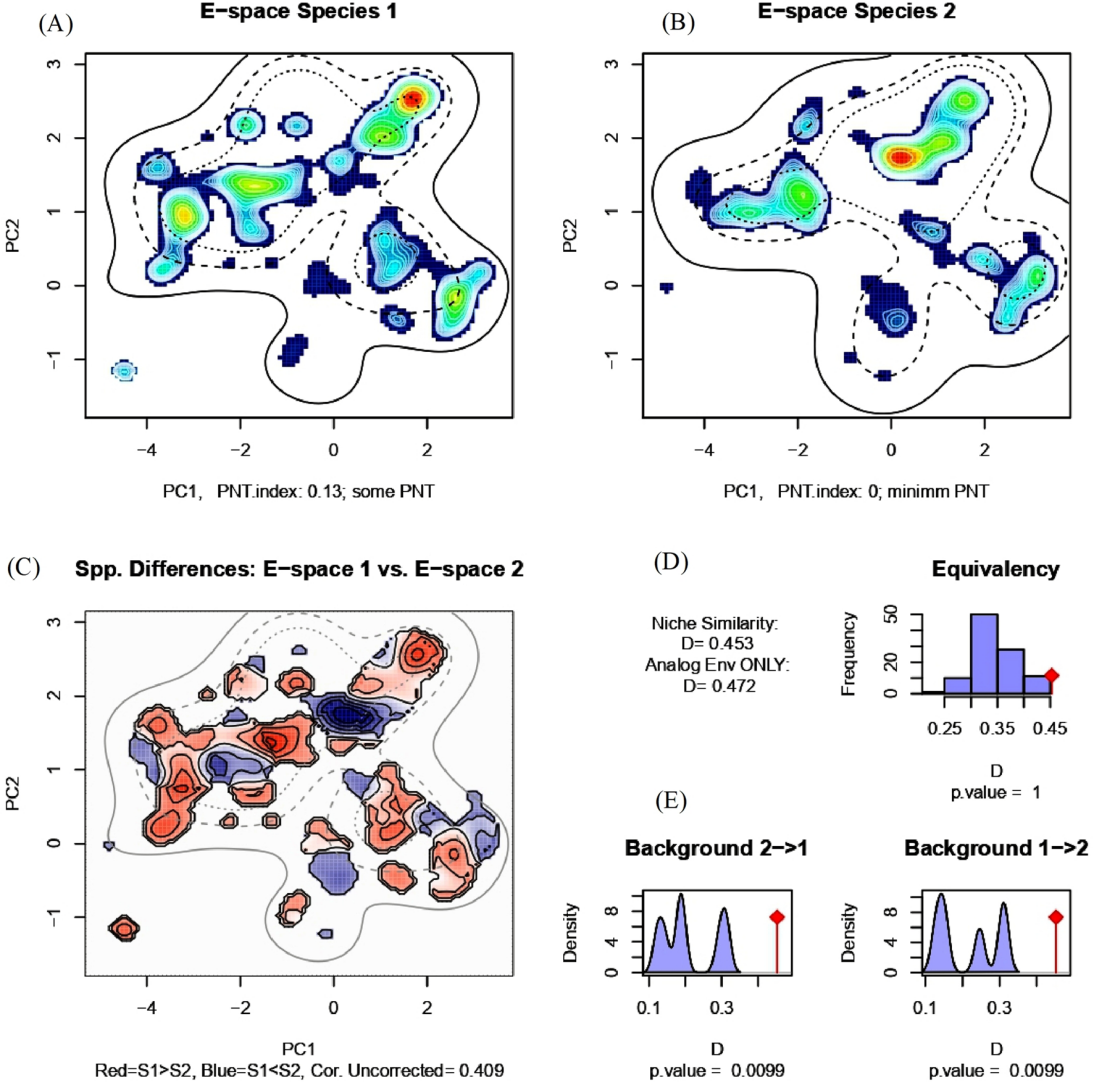
Niche equivalency and niche background tests between tiger and leopard. Panels (**A**) and (**B**) represent the kernel density isopleths, red color indicates high density and cooler color (blue) indicates low density. Panels (**A**) and (**B**) also represent the Potential Niche Truncation (PNT) Index describing the amount of observed E-space of the species that is truncated by the available E-space. Panel (**C**) represents the difference in the E-space of two species and Niche E-space Correlation Index. Panel (**D**) represents the Equivalency statistic measured as Niche similarity index and panel (**E**) represents niche Background statistic.

The PCA analysis revealed that 71.9% of the variance (PC1 = 52.5% and PC2 = 19.4%) in environmental data input can be represented in a 2 dimensional E-space (Fig. 7). The dimension with the most explained variance is plotted on horizontal axis of the PCA correlation circle and second most explanatory variables are plotted on vertical axis of the PCA plot (Fig. 7). We obtained a non-significant niche equivalency test statistic (D = 0.45, p = 1) indicating identical environmental niches of tiger and leopard and a significant background test statistic (p = 0.009) (Fig. 8). The significant background test statistic indicates tiger and leopard are more similar than expected by chance. The Potential Niche Truncation Index (PNTI) for tiger and leopard (Fig. 8) falls well below the proposed range of the values associated with either moderate risk (0.15–0.3) or high risks (0.3) that observed niches do not represent the fundamental niches. Thus the observed niches of tiger and leopard represent the fundamental niches of these species.

## Discussion

Machine learning algorithms are relatively more flexible, accurate, and often require less time than traditional approaches. When using random forest, there is no limitation on the number of predictor variables used compared to the traditional approaches. More traditional approaches such as general linear models, and occupancy approaches depend on technical expertise to meet statistical assumptions to produce the unbiased output. However, more accurate distribution maps of the species are needed for the sustainable management of globally threatened species which can be produced using machine learning algorithms. In this study, we explore the applicability of random forest for building multi-scale distribution maps for tiger and leopard in the human-dominated landscapes of central India. In this study we focused on three important components of the spatial ecology of tigers and leopards: the use of multiple spatial scales in assessing the species habitat relationships, using scale optimized predictor variables to predict the HSMs of tigers and leopards under current and future climatic scenarios and finally determining the patterns of niche conservatism between tigers and leopards. Each of these components is briefly discussed below.

### Scale-dependent habitat selection of tiger and leopard

The scale-dependent habitat relationships among carnivore species have been shown to outperform the approaches of single scale habitat association studies^46–48^. In our multiscale habitat selection study, both the predators responded to most of the habitat variables at broader spatial scales, which are in general agreement with similar studies of other carnivore species at multiple spatial scales. Khosravi et al.^47^ found a strong association of three sympatric carnivores relative to habitat variables at broader spatial scales. Cushman and Wassermann^28^ found American martens responded strongly to predictor variables at broader spatial scales. Our study confirms the findings of the previous studies that large carnivores respond to habitat variables at broader spatial scales.

The relationships between tiger occurrences and human-influenced variables at a small scale reflect the tendency of tigers to avoid anthropogenic pressure in disturbed and human-dominated landscapes at a fine-scale, indicating tigers prefer undisturbed habitats within their immediate vicinities. The broad-scale relationship between sal and sal mix forests and tiger occurrences indicates the importance of dense forest cover for the daily movement and dispersal of tigers at broader scales. Tigers are ambush hunters and cover large distances in search of suitable prey^48^; thus, they need dense forest cover to avoid early detection by prey species^49^. The dense forests of sal and sal associated species may provide ample cover to tigers while covering large distances at broader spatial scales. In contrast, leopards showed relationships with human-influenced variables at broader and medium spatial scales which points towards their broader plasticity or adaptability to anthropogenic factors in human-dominated landscapes at a broader scale. The association of leopards with the habitats at the core-buffer interface reflects the tendency of leopards to occupy edges that represent the high human-use zones.

Our study indicates that habitat specialist species such as tigers tend to occur in less disturbed habitats with thick vegetation cover and need continuous tracts of connected forests at broader spatial scales. The habitat generalist species like leopards due to their broader plasticity and adaptability tend to occur in edges^50–52^ with avoidance of human disturbances at a broader spatial scale.

### Habitat suitability of tigers and leopards under current and future climatic scenarios

In this study, we predicted the suitability of the tiger and leopard relative to scale optimized predictor variables using the random forest algorithm. The predicted suitability maps of tigers and leopards show suitable areas for tigers in dense forests with less human interference and leopards occupied the edges at the core-buffer interface of the reserve (Fig. 5). The dense forests are associated with high prey densities in comparison to disturbed habitats. Chita, sambar and wild pig are some of the most important prey species generally associated with undisturbed habitats and occur more frequently in the diet of tigers^48,53^. Sambar, one of the important prey species, is known to avoid disturbed areas^54,55^ actively. Tigers are ambush hunters and cover large distances in search of suitable prey^48^ thus require dense forest cover to avoid early detection by prey species^56^. The dense sal and sal mix forests may provide ample cover to tigers while covering large distances at broader spatial scales. Thus habitat specialist species such as tigers tend to occur in less disturbed habitats with thick vegetation cover and need continuous tracts of connected forests at broader spatial scales. In contrast, leopards showed high occurrences within disturbed habitats at the core-buffer interface of the reserve reflecting their tendency to occupy edges representing high human-use zones. The habitat generalist species like leopards due to their adaptability tend to occur in the edges^57,58^ with high tolerance to the presence of humans^51,52^.

We observed considerable loss of suitable habitat for tigers under all emission scenarios and overall gain in case of leopard (Table 1). Tigers are habitat specialists preferring the areas with dense vegetation cover, high prey density, and less human footprint^59^. Different species are reported to respond to the impacts of climate change differently^60^. Pandey and Papeş^61^ reported expansion in the habitats of generalist mammalian species under future climatic scenarios. Leopards by their broader niches and ecological plasticity, may cope and adapt to the impacts of future climatic changes better than tigers. Our study agrees with the findings of Tian et al.^62^, who predicted that Amur tigers (*Panthera tigris altaica*) would go extinct fastest in severe climate change scenarios. Some authors argue that leopards due to their globally broad distribution can remain unaffected or even benefit from the impacts of climate change as long as their potential prey species suffice^63^. Our study also predicts the highest loss in the potential habitat of tigers under high emission scenarios (RCP 8.5) and expansion in the habitats of leopards under all emission scenarios. Despite the successful application of climatic models to predict the change in the distribution of wide range of species at large scales, they have been questioned for lacking the details on species interactions and species dispersal capabilities. For example, tiger densities are directly dependent on prey abundances^64^ and thus, the change in the distribution of tigers may also depend on how the prey species would respond to the future climate change. Tigers have great dispersal abilities; however the persistent habitat fragmentation and isolation between the protected areas may negatively affect their populations. Thus in future, the increased inter-patch connectivity and maintaining prey species at high densities may locally increase the tiger sub-populations.

### Environmental niche overlap between tiger and leopard

Although we focused on two measures of niche overlap between tiger and leopard in this study, there are other alternatives which may be better suited for particular studies. We observed moderate niche similarity between tiger and leopard (D = 0.54). The non-significant niche similarity test statistic and significant background test statistic suggest a degree of niche conservation between tiger and leopard. We stress here that in this analysis we have treated niche conservation as the tendency of closely related species to share similar traits. However, despite having somewhat similar environmental niches, tigers and leopards varied in their use of specific environmental resources. For example, the ENMs of tiger and leopard showed that tigers occupied dense sal forests with a low human footprint while as suitable areas for leopards included habitats at core-buffer interface with a high human footprint. Leopards tend to avoid tigers when they co-occur and thus use buffer zones around protected areas in India^51^. Similar results of leopards avoiding tigers spatially are regarded as a mechanism of spatial segregation between them^65–67^. In this study, we assessed the environmental niche overlap between the tiger and leopard along the only spatial dimension. However, the carnivore communities are shaped by complex, competitive interactions. High niche similarity among the competing species in one niche dimension is followed by niche dissimilarity in other niche dimensions^68,69^. Sympatric carnivores may achieve the ecological coexistence by using different habitats^70–72^ selecting different prey sizes^62^ or have non-overlapping activity patterns^70–72^.

### Conclusion and management implications

Our study highlights three key components concerning the spatial ecology of two large carnivores in human-dominated landscapes. Our study shows how two ecologically similar species differ in their use of habitat resources across spatial scales. While tigers perceive human avoidance at a small scale, leopards respond to human interference at a broader scale indicating the low tolerance level in tigers towards humans than leopards. The results of multi-scale HSMs of tigers and leopards indicate the importance of dense forest habitats at a broader scale. The regional and landscape planning to mitigate the impacts of future climate change on the persistence of tigers is thus needed. Increasing the forest cover and inter-patch connectivity by building corridors and maintaining prey species at high densities are particularly important management and conservation strategies that could be undertaken for the persistence of tigers.

## Materials and methods

### Study area

**Figure 9:**
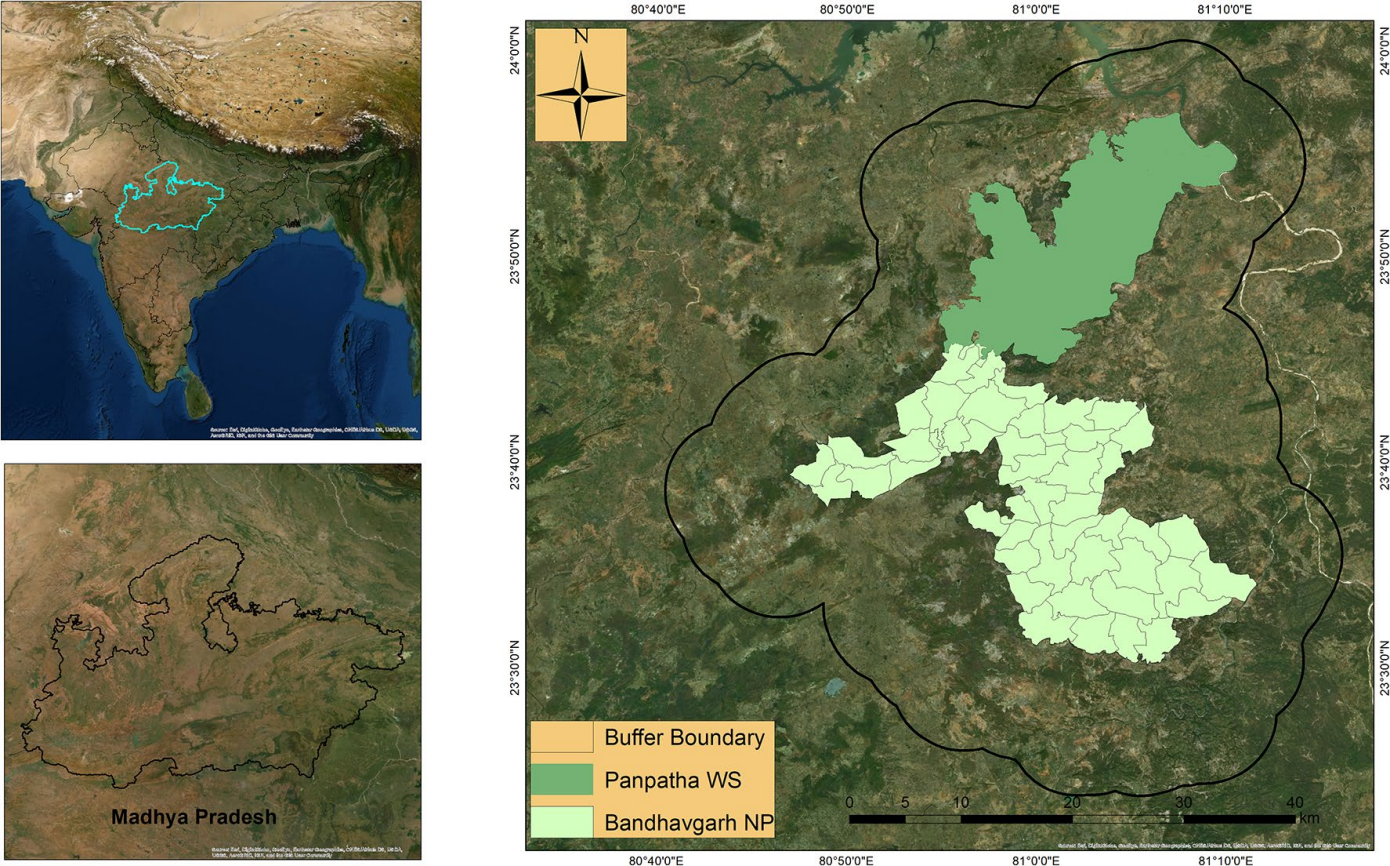
Location of the Bandhavgarh Tiger Reserve, Madhya Pradesh, India. Map of the study area was created using ArcGIS (v 10.3) software developed by ESRI. https://www.esri.com.

**Figure 10:**
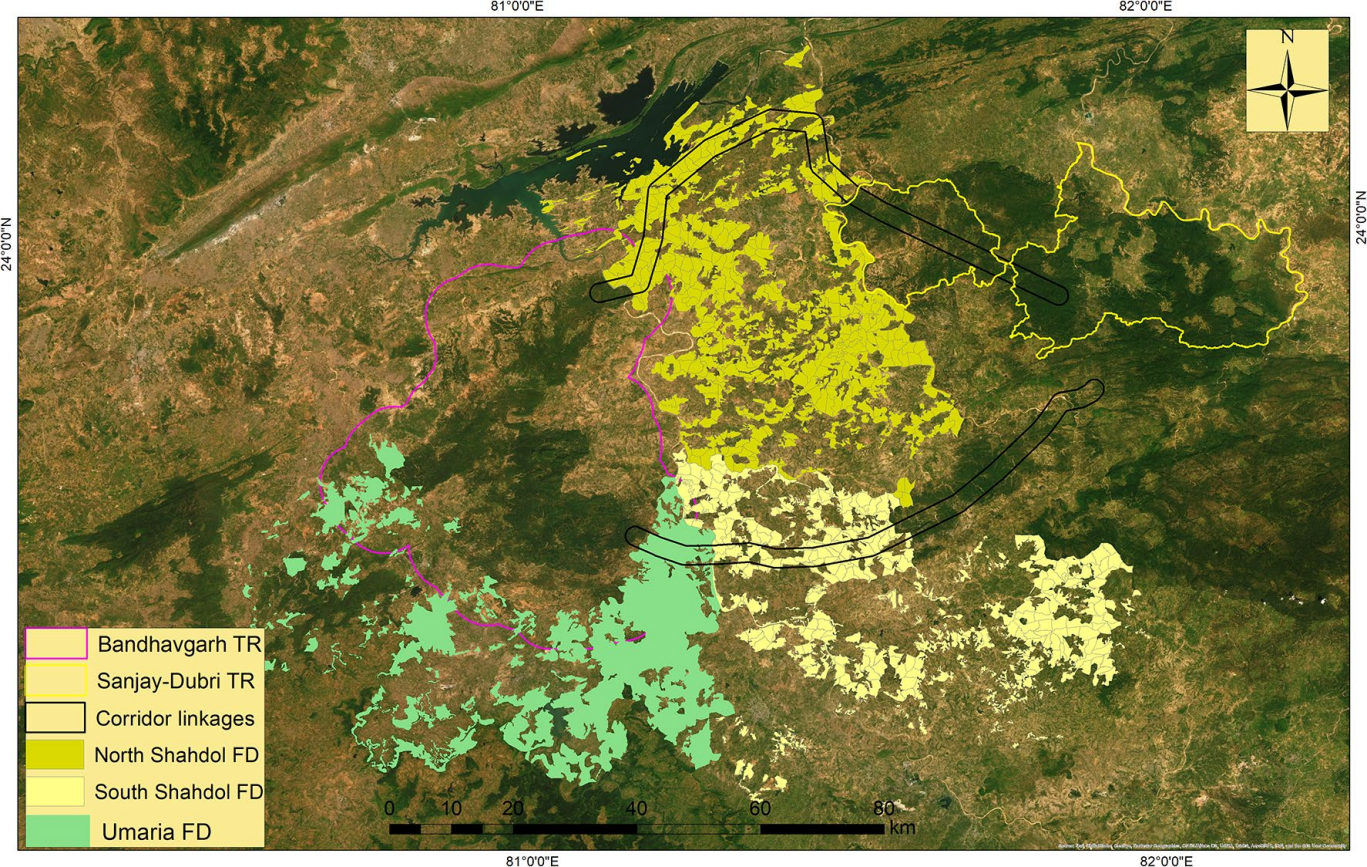
Map showing the Bandhavgarh Tiger Reserve (BTR), Sanjay-Dubri Tiger Reserve (SDTR) in the west, North Shahdol Forest Division (NSFD), South Shahdol Forest Division (SSFD), between BTR and SDTR and Umaria Forest Division (UFD) in south and south east of BTR. The map was created using ArcGIS (v 10.3) software developed by ESRI. https://www.esri.com.

Bandhavgarh Tiger Reserve (BTR) is located between 23° 27′ 00″ to 23° 59′ 50″ North latitude and 80° 47′ 75″ to 81° 15′ 45″ East longitude in the Umaria district of Madhya Pradesh, in central India (Fig. 9). The core zone of the reserve includes the Panpatha Wildlife Sanctuary (PWS) in north and Bandhavgarh National Park (BNP) in the south, together with having an area of 716 km^2^. The surrounding buffer zone has an area of 820 km^2^, adding the total area of the reserve to 1536 km^2^. The reserve is surrounded by the fragmented and human-dominated territorial forest ranges of the North Shahdol Forest Division (NSFD) in the north and northeast and south Shahdol Forest Division (SSFD) in the south-southeast. The territorial forest division of district Umaria (UFD) surround the reserve in extreme south and southwest, and the Katni forest division (KFD) is located to the west of the reserve. The Sanjay-Dubri Tiger Reserve (SDTR) and Guru-Ghasidas Tiger Reserve (GGTR) are located about 80–150 km from the BTR in the northeast and southeast, respectively. The whole landscape (BTR, SDTR, NSFD, SSFD, and GGTR) is regarded as an important tiger and elephant conservation unit (Fig. 10).

BTR represents the moist deciduous vegetation dominated by sal (*Shorea robusta*) and sal mixed forests. The overall vegetation of the BTR comprises moist peninsular low-level sal forest, northern dry mixed deciduous forest, dry deciduous scrub, dry grassland and west Gangetic moist mixed deciduous forest^73^. BTR supports a wide variety of faunal assemblages from small invertebrates to the largest bovid in Asia. There are 35 mammalian species, over 250 species of birds, and a wide variety of butterflies in reserve. The deer species include chital (*Axis axis*), sambar (*Rusa unicolor*) and barking deer (*Muntiacus munjtak*). Indian gazelle (*Gazella bennetti*), four-horned antelope (*Tetracerus quadricornis*) and Indian blue bull (*Boselaphus tragocamelus*) are the three antelope species in BTR. Northern plains gray langur (*Semnopithecus entellus*) and rhesus macaque (*Macaca mulatta*) represent the two primate species, and the suidae family is represented by a wild pig (*Sus scrofa*). The reserve also holds a good population of re-introduced gaur (*Bos gaurus*).

Major large carnivore species include tiger (*Panthera tigris*), leopard (*Panthera pardus*), sloth bear (*Melursus ursinus*), Indian wolf (*Canis lupus*), Asiatic wild dogs (*Cuon alpinus*) and striped hyena (*Hyaena hyaena*). Golden jackal (*Canis aureus*), Indian fox (*Vulpes bengalensis*), jungle cat (*Felis chaus*), Asiatic wildcat (*Felis lybica ornata*), rusty-spotted cat (*Prionailurus rubiginosus*) and fishing cat (*Prionailurus viverrinus*) are the medium-sized carnivores in reserve.

### Species occurrence data and spatial auto-correlation

The species occurrence data was obtained by collecting the scats of tigers and leopards in the study area. We collected 381 and 343 scats of tigers and leopards between 2017 and 2018. The identification of the scats was based on secondary evidence such as diameter range, and presence of associated ancillary signs like tracks^74,75^. Andheria et al.^76^ confirmed the accuracy of scat identification using the same features with fecal DNA tests. The scats where the identity of the predator was ambiguous were not collected. We obtained an additional 95 and 74 camera trap detection of tigers and leopards in a camera trap survey in the buffer zone of the reserve. We implemented spatial filtering using the SDM toolbox^77^ in ArcGIS (10.3) to reduce the inherent spatial bias in the species presence records. The scats and camera trap photo-captures of tigers and leopards were spatiality rarified at the distance of 1,000 m from each other. Lacking the real absence points, we randomly generated pseudo-absence points in ArcGIS (10.3) in an approximately equal number to the original occurrence points of the tiger and leopard to deal with the problems arising from unbalanced prevalence^78^. This was achieved by first generating 500 random pseudo absence points and then discarding the absence points within the buffer radius of 500 m of the original occurrence points of tiger and leopards to reduce the number of false negatives^79^. The buffer distance can be either set arbitrary or based on species attributes^80^. Out of 476 and 417 occurrence records for tigers and leopards, we retained a total of 184 and 261 spatially rarified occurrence locations and an equal number of pseudo absence points of tigers and leopards for final random forest modeling.

### Environmental predictors

A total of 40 environmental predictor variables (Table 2) were used in predicting the species habitat relationships. We grouped predictor variables into five broad categories as climatic, topographic, landscape composition, vegetation, and human-influenced.

**Table 2:** The set of 40 predictor variables used in the multi-scale habitat modelling of tiger and leopard. First and second columns represent the type and the name of the variables, and third and fourth columns represent scale at which each predictor best explained the occurrence of tiger and leopard respectively.

Variable Type	Variable	Best scale (tiger)	Best scale (leopard)
Topographic	Elevation	24,500	24,500
	Slope	17,500	14,000
	Aspect	3,500	28,000
	Terrain roughness	24,500	17,500
	River density	1,000, 2,000, 3,000	1,000, 2,000, 3,000
Climatic	Bio1	28,000	24,500
	Bio2	28,000	24,500
	Bio3	17,500	24,500
	Bio4	7,000	21,000
	Bio5	21,000	7,000
	Bio6	21,000	14,000
	Bio7	21,000	7,000
	Bio8	28,000	24,500
	Bio9	28,000	24,500
	Bio10	28,000	28,000
	Bio11	14,000	21,000
	Bio12	7,000	24,500
	Bio13	7,000	3,500
	Bio14	14,000	10,500
	Bio15	28,000	28,000
	Bio16	7,000	28,000
	Bio17	28,000	28,000
	Bio18	24,500	7,000
	Bio19	17,500	17,500
	Actual evapotranspiration (summer)	10,500	NA
	Actual evapotranspiration (monsoon)	17,500	NA
	Actual evapotranspiration (winter)	NA	NA
Landscape composition	Sal dominated	28,000	21,000
	Sal mix	28,000	21,000
	Dry deciduous	24,500	28,000
	Moist deciduous	28,000	14,000
	Degraded	3,500	24,500
	Scrub	28,000	21,000
Vegetation cover	NDVI (summer)	7,000	14,000
	NDVI (winter)	21,000	17,500
	NDVI (monsoon)	28,000	10,500
Human influenced	Human settlements	7,000	28,000
	Human population density	3,500	21,000
	Road density	1,000, 3,000, 4,000	1,000, 3,000, 4,000
	Farmlands (croplands)	7,000	10,500

Predictor variables are classified in five groups (Topographic, climatic, landscape composition, vegetation and human influenced). Road and river density were calculated at four different spatial scales (1, 2, 3, 4 km). Actual evapotranspiration were not used in the models of leopard and in case of tiger, actual evapotranspiration in winter season was not used.

**Figure 11:**
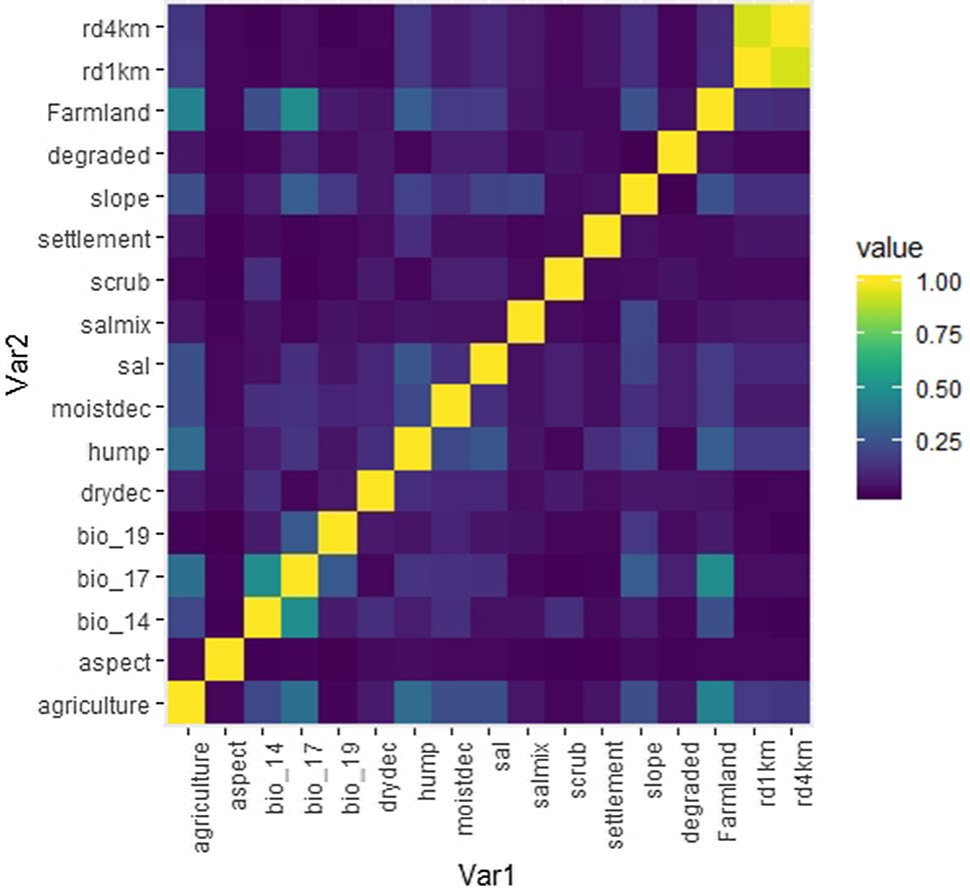
Multi-colinearity among the predictor variables used in the final random forest modelling of tiger and leopard. The multi-colinearity was tested at (r > 0.50) using the R package “rfUtilities” and correlogram was produced using the R package “ENMTools”. The road density is represented at two spatial scales (1 km and 4 km) as shown in the top right corner of the correlogram as rd1km and rd4km, and moistdec, drydec represent the moist and dry deciduous forests respectively and hump denotes the human population density defined as the number of persons per square km.

These predictor variables were selected based on the similar habitat relationship studies of large carnivores. We obtained the bioclimatic variables from the WORLDCLIM database (https://www.worldclim.org). We tested the correlation among predictor variables at (|r| > 0.50) and subsequently removed the highly correlated variables using R packages “rfUtilities” and “randomForest”^81^ implemented in R^82^ to account for multi-colinearity among predictor variables^83^ as multi-colinearity may alter the model predictions to the significant extent^84^. The package “rfUtilities” removes the redundant variables using qr matrix decomposition (0.05 threshold) and thus only the least correlated variables (|r| < 0.50) were retained for further modelling (Fig. 11). We obtained a digital elevation map of the study area from the Shuttle Radar Topography Mission (SRTM) elevation database^85,86^. Slope, aspect, topographic ruggedness index was derived from the elevation layer using surface analysis tools in the Spatial Analyst toolbox in ArcGIS (10.3). We obtained the land use land cover (LULC) from the Indian Institute of Remote Sensing (IIRS, https://iirs.gov.in). The LULC layer included nine land use categories as dense sal dominated forests, sal mix forests, moist deciduous forests, dry deciduous forests, scrub habitats, grasslands, agriculture, water bodies and permanent human settlements. We calculated seven topographic variables including elevation, slope, aspect, topographic roughness, road density and river density. We calculated road and river density using the line density tool in ArcGIS at the spatial scale of 1,000, 2,000, 3,000 m. We used road and river density instead of Euclidean distance because of the high concentration of roads in the buffer zone of the reserve. Thus we used the percentage of roads and rivers within the radius 1,000, 2,000, and 3,000 m of the specie presence-absence. Monthly Normalized Difference Vegetation Index (NDVI) version 6 (MOD13Q1) generated every 16 days available at the spatial resolution of 250 m was obtained from the MODIS website (https://lpdaac.usgs.gov/products/mod13q1v006/). We reclassified the 23 NDVI layers into three seasons corresponding to summer, wet, and winter seasons. We resampled all the variables at the spatial resolution of 90 m using the SDM toolbox in ArcGIS (10.3).

### Future climatic data

At present, climatic models are the best tools for simulating future climatic scenarios^88^. However, the variations within and among the different climatic models may pose problems in identifying the most robust and optimal model to use^88^. Though, no clear guidance on how and which climatic models to select exists, and researchers have little objectivity in selecting the climatic models^87^. The final decision may sometimes be influenced by the assumption (though not always correct) that a climatic model developed in a particular country will be more robust in that region^87^. In this study, we modeled the change in the potential distribution of tigers and leopards under two Representative Concentration Pathway Scenarios (RCP 2.6 and RCP 8.5) developed by the Japanese research community called Model for Inter-disciplinary Research on Climate change (MIROC5)^89^. These scenarios project the global greenhouse gas emissions based on the assumptions for a wide range of variables such as human population size, global energy consumption, and change in land-use patterns. We downloaded Global Climate Models (GCMs) from the WordClim website (https://www.worldclim.org/cmip5_30s). We aimed to predict the change in the distribution under the most conservative emissions scenario (closely corresponding to the current rate of greenhouse gas emissions) and the most severe emission scenarios. The climatic models used in this study represent two extreme scenarios of greenhouse gas emissions. The RCP 2.6 assumes that global CO_2_ emissions would peak around 2020 and then fall to values around zero by 2080 and RCP 8.5 is regarded as the worst climatic scenario with higher predicted greenhouse gas emissions. RCP 8.5 assumes that the global CO_2_ emissions would increase at a higher rate during the first half of the century and stabilize by 2100; the concentrations are however three times those in 2000^90–93^.

### Multi-scale data processing

We calculated the focal mean of each predictor variable across eight spatial scales (3,500–28,000 m) surrounding each species occurrence location (presence/pseudo absence) using a moving window analysis with the focal statistic tool in ArcGIS (10.3). Each spatial scale ranging from 3,500 m to 28,000 m surrounding each location was used as search radii for calculating the focal mean of all the predictor variables expect road and river density. The output of the focal statistics was the raster layers of each predictor variable at eight spatial scales and .dbf file of extracted raster values around each location of tigers and leopards.

### Scale selection and univariate random forest models

The best predictive scale in multi-scale modeling approaches is usually selected by measuring potential environmental predictor variables within different buffer sizes (scales) around species locations (presence/absence) and then to regress each predictor variable against the response for each scale^94,95^. Following McGarigal et al.^96^ and Cushman et al.^34^, we ran a series of univariate random forest models for each predictor variable across eight spatial scales (3,500–28,000 m) to select the appropriate scale at which the predictor variable best explained the probability of species occurrence. The univariate random forest models were run with the underlying assumption that best fit identifies the most predictive, and therefore, the single most meaningful, spatial scale across all predictor variables^15^.

Random forest constructs a regression or classification tree by successively splitting the data based on single predictors. Each split forms a branch in the decision tree and trees are grown without pruning. Random forest utilizes bagging (bootstrap aggregation) that builds a large number of tress and the model output is obtained by averaging the aggregated tress or by maximum vote. During bagging, a bootstrap sample is randomly drawn to build each tree and the data not included in the bootstrap sample is termed as ‘out-of-bag’ (OOB) which is used to estimate an unbiased error rate and to rank variable importance. We used OOB rates as a measure of selecting the best predictive spatial scale. In the calculation of OOB error rates, a training data set is created by sampling with replacement from two-third of the data for each classification tree in a random forest. Each tree is then used to predict the remaining one-third (‘out of bag’ or ‘bootstrap sample’) of the data. Finally, the OOB error is computed as the proportion of times that the predicted class is not the same as the true class^26,81^. The scale with the minimum OOB error rates was selected as the best spatial scale of the predictor variables.

### Multi-scale random forest modeling

Multi-scale random forest models were created using the scale optimized predictors of tigers and leopards with R package ‘randomForest’^81^ implemented in R^82^. Model Improvement Ratio (MIR) was used to identify the most parsimonious random forest model. In the model selection process using MIR, the variables were subset using 0.10 increments of MIR values, and all variables above this threshold were retained for each model. This subset was always performed on the original model’s variable importance to avoid overfitting. Comparisons were made between each subset model, and the model with the lowest OOB error rate and lowest maximum within-class error was selected as the final model.

### Random forest variable selection

There are several variable selection procedures available in random forest^97–99^ we followed the approach of variable selection developed by Genuer et al.^99^. This approach is based on the un-scaled permutation importance that is calculated by permuting each predictor in turn and using the difference in prediction error (OOB error) before and after permutation as a measure of variable importance^15,81,100^. This approach is a stepwise procedure whereby a sequence of RF models is estimated by iteratively eliminating or adding variables according to their importance measures (such as MIR)^101^. The MIR shows variable importance measured as the increased mean square error (%IncMSE), which represents the deterioration of the predictive ability of the model when each predictor is replaced in turn by random noise. Higher % IncMSE indicates greater variable importance. In this way, we selected only those variables that improved model performance.

### Model assessment

We used AUC (area under the receiver operating characteristic curve) ROC and True Skill Statistic (TSS) as a means of model performance. Ponitus and Milones^102^ reported that Kappa Statistics does not provide a meaningful statistical measure of predictive success. Thus, we avoided the use of Kappa Statistics as a measure of model performance. Ponitus and Si^103^ also argue that transforming the continuous predicted probabilities of a predictive model into binary response requires the use of certain threshold cut-point values, which makes the actual quality of prediction less informative. Cushman and Wasserman^28^ while comparing the multi-scale habitat selection of American martens using logistic regression and random forest also recommend the use of AUC instead of Kappa Statistics as a measure of model performance. Models with AUC values of 0.7–0.9 are considered useful whereas the values higher than 0.9 are regarded as models with excellent discrimination abilities or high predictive power^94,95^.

### Multiscale random forest distribution maps

Following the procedures of univariate random forest models (scale optimization), selection of important variables, and model assessment, we used scale optimized variables to predict the final distribution maps of tiger and leopard using the R package ‘randomForest” in R^81^. The future distribution maps of the tiger and leopard were predicted using the same scale optimized variables expect, the bioclimatic variables corresponding to the greenhouse gas emission scenarios (RCP 2.6 and RCP 8.5) were used in future prediction maps for the years the 2050s and 2070s.

### Niche identity and niche background tests

Methods to quantify and test the environmental niche similarities rely either on ordination techniques^104^ or environmental niche models (ENMs)^105^. We used ENMs of tiger and leopard (Fig. 2) to perform Schoener’s niche equivalency (identity) test (D) and Warren’s niche background test (I)^37^ using the R package ‘Humboldt’^44^. Niche equivalency is a one-tailed statistical test used to test out the null hypothesis that two species have identical environmental niches. The niche equivalency test compares the observed niche similarity between the ENMs of two species and a niche background test assesses the power to detect the differences between the ENMs of two species. The values of niche similarity (D) range from 0 indicating complete dissimilar niches to 1 indicating complete similar niches^37,44^. The statistics calculate how similar the occupied niches of two species are to each other based on original input occurrences by calculating the Schoener’s D. The observed values of Schoener’s D are then compared to the indices obtained by resampling and reshuffling the species occurrence locations. At each resampling, the occurrences of species 1 and species 2 are pooled and then assigned randomly to one of the two groups. At each iteration, the Schoener’s D and Warren’s I are measured between any two reshuffled groups. The actual observed values of Schoener’s D and Warren’s I based on original occurrences are then compared with the null distribution created from all the values obtained from the reshuffled occurrences. Thus background test compares the observed niche similarity based on original occurrence locations between species 1 and species 2 to the overlap generated between species 1 and the random shifting of the spatial distribution of species 2 in geographic space and then measuring how this shift in geography changes occupied environmental space. In brief, the background test determines if the two distributed species are more different than would be expected given the underlying environmental differences between the habitats in which they occur.

A non-significant equivalency statistic and a significant background statistic support the underlying null hypothesis that species environmental niches are identical. A statistically significant equivalency statistic, regardless of the significance of background statistics, results in the rejection of the null hypothesis of niche equivalency^44^. If both the equivalency statistic and background statistic are statistically non-significant, it implies that observed niche similarity is a result of space limitations and that there is a low power for the equivalence statistic to detect the meaningful and significant differences among the species niches^44^.

### Realization of species fundamental niche from observed niche

The identification of species' fundamental niche from the species occupied niche remains one of the major challenges in the studies of niche analysis^106^. Most studies usually overlook how bad or how well a species occupied niche can reflect the species' fundamental niche. The package ‘Humboldt’ provides a way to characterize the fundamental niche by truncating species occupied E-space by the available E-space in its environment. There is a directly proportional relationship between the portion of the occupied niche in E-space truncated and the risk that occupied niche poorly represents the species fundamental niche. Thus higher the proportion truncated, greater the risk that occupied niche poorly reflects the species fundamental niche. Brown and Carnaval^44^ introduced the concept of the Potential Niche Truncation Index (PNTI) implemented in package ‘Humboldt’ which quantifies the amount of observed E-space truncated by the available E-space. It specifically measures the overlap between the 5% kernel density isopleths of species E-space and the 10% density isopleths of the available or accessible E-space in the environment. This value is the realization of how much of the perimeter of the species E-space abuts, overlaps or falls outside the margins of the environment’s E-space. The values of PNTI in the range of (0.15–0.3) have moderate risks and the values greater than (0.3) have a high risk that observed niches do not represent the fundamental niches due to niche truncation driven by limited available E-space^44^.

## Data availability

The data sets that were generated or analysed in this study are included in the supplementary information. The R codes used in the multiscale habitat analysis can be obtained on a request from the corresponding author.

## Supplementary information

Supplementary Information 1.

Supplementary Information 2.

Supplementary Information 3.

Supplementary Information 4.
