# Supplementary material for: Multi-scale habitat modelling and predicting change in the distribution of tiger and leopard using random forest algorithm
Source: Sci Rep. 2020 Jul 10;10:11473. doi: 10.1038/s41598-020-68167-z (PMC7351791; doi:10.1038/s41598-020-68167-z)
Supplement: Supplementary file 3 — Supplementary Information 3. [file 41598_2020_68167_MOESM3_ESM.docx]

**Supplementary S3: R code used to generate the niche overlap maps using package 'humboldt'**

**library(devtools)**

**install_github("jasonleebrown/humboldt")**

**library(humboldt)**

**library(raster)**

**setwd("E:/Humboldt/ascii")**

**agri<- raster("agri24500_tif.asc")**

**aspect<- raster("aspect28000_tif.asc")**

**bio14<- raster("bio14_14000_tif.asc")**

**bio17<- raster("bio17_28000_tif.asc")**

**degraded<- raster("degraded24500_tif.asc")**

**hump<- raster("hump3500_tif.asc")**

**moistdec<- raster("moistdec14000_tif.asc")**

**road1km<- raster("road1km_tif.asc")**

**sal<- raster("sal28000_tif.asc")**

**salmix<- raster("salmix21000_tif.asc")**

**scrub<- raster("scrub21000_tif.asc")**

**settlement<- raster("settlement7000_tif.asc")**

**slope<- raster("slope14000_tif.asc")**

**env <- stack(agri,aspect,bio14,bio17,degraded,**

**hump,moistdec,road1km,sal,salmix,scrub,**

**settlement,slope)**

**env <- setMinMax(env)**

**occ<- read.csv("leo_loc.csv")**

**rasValue=extract(env, occ)**

**combinePointValue=cbind(occ,rasValue)**

**write.csv(combinePointValue, "env1.csv")**

**write.csv(combinePointValue, "sp1.csv")**

**occ2<- read.csv("tig_loc.csv")**

**rasValue=extract(env, occ2)**

**combinePointValue2=cbind(occ2,rasValue)**

**combinePointValue2**

**write.csv(combinePointValue2, "env2.csv")**

**write.csv(combinePointValue2, "sp2.csv")**

**env1<-humboldt.scrub.env(read.csv("E:/Humboldt/ascii/env1.csv"))**

**env2<-humboldt.scrub.env(read.csv("E:/Humboldt/ascii/env2.csv"))**

**sp1<- read.csv("E:/Humboldt/ascii/sp1.csv")**

**sp2<- read.csv("E:/Humboldt/ascii/sp2.csv")**

**reduc.vars<- humboldt.top.env(env1=env1,env2=env2,**

**sp1=sp1,sp2=sp2,**

**rarefy.dist=1,**

**rarefy.units="km",**

**env.reso=0.08,**

**learning.rt1=0.001,learning.rt2=0.001,**

**e.var = c(3:10),**

**pa.ratio=4,steps1=10,**

**steps2=10,method="contrib",contrib.greater=5)**

**num.var.e<-ncol(reduc.vars$env1)**

**num.var.e**

**full<-humboldt.doitall(inname="full_extent",env1=env1, env2=env2,**

**sp1=sp1, sp2=sp2,**

**rarefy.dist=1,**

**rarefy.units="km",**

**env.reso=0.08,**

**reduce.env=2,**

**reductype="PCA",**

**non.analogous.environments="YES",**

**correct.env=T,**

**env.trim=F,**

**env.trim.type="RADIUS",**

**trim.buffer.sp1=1,**

**trim.buffer.sp2=1,**

**pcx=1, pcy=2,**

**col.env=e.var,**

**e.var = c(3:10),**

**color.ramp = 1,**

**R=100,**

**kern.smooth=1,**

**e.reps=100,**

**b.reps=100,**

**nae="YES",**

**thresh.espace.z=0.001,**

**p.overlap=T,**

**p.boxplot=T,**

**p.scatter=T,**

**run.silent=F,**

**ncores=2)**
