# Supplementary material for: Multi-scale habitat modelling and predicting change in the distribution of tiger and leopard using random forest algorithm
Source: Sci Rep. 2020 Jul 10;10:11473. doi: 10.1038/s41598-020-68167-z (PMC7351791; doi:10.1038/s41598-020-68167-z)
Supplement: Supplementary file 4 — Supplementary Information 4. [file 41598_2020_68167_MOESM4_ESM.docx]

**Multi-scale habitat modelling and predicting change in the distribution of tiger and leopard using random forest algorithm**

**Tahir A Rather^*, 1, 2^, Sharad Kumar^1, 2^ and Jamal A Khan^1^**

**^1^Department of Wildlife Sciences, Aligarh Muslim University, Aligarh, Uttar Pradesh, India, 202002**

**^2^The Corbett Foundation, 81-88, Atlanta Building, Nariman Point, Mumbai, 400021, Maharashtra, India**

**Supplementary S1: The presence absence data and a suite of 40 scale optimized predictor variables used in the multi-scale habitat modelling of tiger in Bandhavgarh Tiger Reserve, Madhya Pradesh, India**

**Supplementary S2: The presence absence data and a suite of 40 scale optimized predictor variables used in the multi-scale habitat modelling of leopard in Bandhavgarh Tiger Reserve, Madhya Pradesh, India**

**Supplementary S3: R code used to generate the niche overlap maps using package 'humboldt'**
